# Mutational hotspots in the *TP53 *gene and, possibly, other tumor suppressors evolve by positive selection

**DOI:** 10.1186/1745-6150-1-4

**Published:** 2006-01-31

**Authors:** Galina V Glazko, Vladimir N Babenko, Eugene V Koonin, Igor B Rogozin

**Affiliations:** 1 Department of Biostatistics and Computational Biology, University of Rochester Medical Center, 601 Elmwood Avenue, Rochester, NY 14642, USA; 2 National Center for Biotechnology Information, National Library of Medicine, National Institutes of Health, Bethesda, MD 20894, USA

## Abstract

**Background:**

The mutation spectra of the *TP53 *gene and other tumor suppressors contain multiple hotspots, i.e., sites of non-random, frequent mutation in tumors and/or the germline. The origin of the hotspots remains unclear, the general view being that they represent highly mutable nucleotide contexts which likely reflect effects of different endogenous and exogenous factors shaping the mutation process in specific tissues. The origin of hotspots is of major importance because it has been suggested that mutable contexts could be used to infer mechanisms of mutagenesis contributing to tumorigenesis.

**Results:**

Here we apply three independent tests, accounting for non-uniform base compositions in synonymous and non-synonymous sites, to test whether the hotspots emerge via selection or due to mutational bias. All three tests consistently indicate that the hotspots in the *TP53 *gene evolve, primarily, via positive selection. The results were robust to the elimination of the highly mutable CpG dinucleotides. By contrast, only one, the least conservative test reveals the signature of positive selection in BRCA1, BRCA2, and p16. Elucidation of the origin of the hotspots in these genes requires more data on somatic mutations in tumors.

**Conclusion:**

The results of this analysis seem to indicate that positive selection for gain-of-function in tumor suppressor genes is an important aspect of tumorigenesis, blurring the distinction between tumor suppressors and oncogenes.

**Reviewers:**

This article was reviewed by Sandor Pongor, Christopher Lee and Mikhail Blagosklonny.

## Open peer review

Reviewed by Sandor Pongor, Christopher Lee and Mikhail Blagosklonny.

For the full reviews, please go to the Reviewers' comments section.

## Background

The p53 protein is called "the guardian of the genome" because this multifunctional transcription factor, which regulates cell cycle progression, repair and programmed cell death in mammals, targets for apoptosis those cells that accumulate unsustainable DNA damage [1-4]. In nearly 60% of human cancers, the *TP53 *gene carries mutations that are generally thought to abrogate the tumor suppressor function of p53 [1-4]. However, many independent studies have also revealed gain of new biochemical and biological functions as a result of *TP53 *mutations, suggesting that this gene additionally has properties of an oncogene [4-10]. Recent reports on mouse models of the Li-Fraumeni syndrome (LFS), a familial cancer predisposition syndrome caused by germline p53 mutations, revealed significant changes in the tumor spectra in mice carrying common p53 mutations, indicating that gain-of-function in p53 is important for tumorigenesis [11,12].

This notion has been supported and extended by bioinformatic analysis of the tumor-specific mutation spectra in the *TP53 *gene which show a highly significant excess of non-synonymous mutations over the neutral expectation, suggesting that p53 evolution in tumors is subject to positive selection [13] as a result of preferential fixation of missense mutations in p53 [14-16].

However, this apparent positive selection does not necessarily account for the strongly non-uniform distribution of mutations among the sites in the *TP*53 gene, i.e., the existence of hotspots. The presence of hotspots is compatible with either a mutational or a selectional scenario or a combination thereof [14,17]. A well-characterized case of apparent mutational origin of hotspots is the preponderance of G>T transitions in the *TP53 *gene in lung cancers which is usually perceived as a reflection of the mutagenesis specificity of polycyclic carcinogens [18,19]. However, this paradigm has been challenged on the grounds that the respective mutations localize predominantly in highly conserved, functionally important sites of p53 [20]. It is important to determine the relative contributions of selection and mutation specificity to the hotspot origin because it has been suggested that mutable nucleotide contexts could be used to infer mechanisms of mutagenesis and thus elucidate key mechanisms of tumor initiation and progression [18,19]. This type of analysis has proved informative for somatic hypermutation in immunoglobulin genes [21,22], some cancer-related genes in lymphomas [23], and germline mutations in human disease genes [24,25].

Here we apply three independent tests, accounting for non-uniform base compositions in synonymous and non-synonymous sites, to test whether selection makes a significant contribution to the origin of the hotspots. All three tests consistently indicate that the hotspots in the *TP53 *gene evolve, primarily, via positive selection. By contrast, only one, the least conservative test reveals the signature of positive selection in BRCA1, BRCA2, and p16. The results of this analysis seem to indicate that positive selection for gain-of-function in tumor suppressor genes is an important aspect of tumorigenesis, blurring the distinction between tumor suppressors and oncogenes.

## Results and discussion

Many independent studies have revealed gain of new biochemical and biological functions as a result of *TP53 *mutations, suggesting that this gene additionally has properties of an oncogene [4-12]. This notion has been supported by bioinformatic analysis of the tumor-specific mutation spectra in the *TP53 *gene which show a highly significant excess of missense mutations over the neutral expectation, suggesting that p53 evolution in tumors is subject to positive selection [14,15]. In Table 1, we present an update of this comparison based on the latest somatic mutation data; the substantial excess of non-synonymous substitutions suggests positive selection [13] acting on p53 in all tumor types for which sufficient information was available.

**Table 1:** NSMC and NSCS test results for *TP53 *somatic mutation spectra (*H0*: mutational bias; *H1*: selectional bias)

	Sites	Mutations	Hotspots	P(*H1*)/χ^2^
**Bladder**				
Synonymous	61	80	12	
Non-synonymous	240	775	110	0.107/***13.8***
Nonsense	36	85	26	0.038
**Brain**				
Synonymous	20	23	2	
Non-synonymous	199	812	98	***0.998/11.2***
Nonsense	14	32	5	0.015
**Breast**				
Synonymous	77	108	19	
Non-synonymous	294	1423	171	0.111/***27.3***
Nonsense	49	73	27	0.052
**Liver**				
Synonymous	34	43	5	
Non-synonymous	199	727	99	***0.980/14.4***
Nonsense	29	50	16	0.040
**Lung**				
Synonymous	56	88	17	
Non-synonymous	314	1599	191	***0.959/17.9***
Nonsense	55	170	31	0.042
**Pancreas**				
Synonymous	10	12	1	
Non-synonymous	106	215	30	0.049/1.56
Nonsense	10	17	6	0.058
**Ovary**				
Synonymous	20	21	1	
Non-synonymous	192	770	99	***0.995/15.7***
Nonsense	30	71	12	0.012
**Prostate**				
Synonymous	28	38	7	
Non-synonymous	114	195	38	0.926/0.72
Nonsense	9	12	2	0.008
**Colon**				
Synonymous	23	28	4	
Non-synonymous	173	592	69	***1.000/4.4***
Nonsense	19	60	5	0.016
**Colorectal**				
Synonymous	25	28	4	
Non-synonymous	203	1097	121	0.000/***20.3***
Nonsense	29	103	12	***0.953***
**Esophagus**				
Synonymous	25	32	7	
Non-synonymous	215	1064	117	0.025/***6.26***
Nonsense	50	150	27	0.032
**Hematopoietic**				
Synonymous	26	29	3	
Non-synonymous	202	723	99	0.037/***13.08***
Nonsense	20	37	5	0.018
**Larynx**				
Synonymous	8	10	2	
Non-synonymous	108	234	42	***1.000/***0.60
Nonsense	18	24	5	0.007
**Mouth**				
Synonymous	28	41	9	
Non-synonymous	176	413	79	***0.988/***1.59
Nonsense	33	53	10	0.901
**Skin**				
Synonymous	64	136	12	
Non-synonymous	248	563	99	***0.931/9.94***
Nonsense	25	69	12	0.025
**Stomach**				
Synonymous	44	64	15	
Non-synonymous	195	617	88	0.115/1.78
Nonsense	21	52	8	0.029

In the last column, the P-value for the NSMC test (numerator) and the χ^2 ^value for the NSCS test (denominator) are shown along with the NSMC test P-value for the hotspots in nonsense sites. The statistically significant values (rejection of *H0*) are shown in bold type.

**Figure 1:**
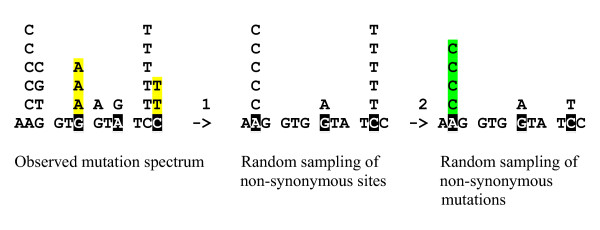
**The procedure used for random sampling of mutations at non-synonymous sites in the NSMC test**. Step 1 includes the selection of a sample of non-synonymous sites such that the number of sites and their base composition were the same as in the entire set of synonymous substitutions in the given gene; sampling was performed without replacement. The comparison of the number of hotspots, i.e., sites with at least two substitutions, between the samples of synonymous and non-synonymous sites gives a measure of the skewness of the distribution of mutations (Step 2). Synonymous sites and sampled non-synonymous sites are shown by inverse shading, synonymous hotspots are shown in yellow, and simulated non-synonymous hotspots are shown in green.

We developed a simple statistical test (hereinafter NSMC test, after Non-Synonymous Monte Carlo) that specifically addressed the dilemma of mutational vs. selectional origin of the hotspots. This test included comparison of samples of synonymous and non-synonymous sites selected such that both the number of sites and the number of mutations in the samples was the same (Fig. 1). Only positions in which mutations were found were analyzed. Sites in which both synonymous and non-synonymous substitutions were observed (e.g., third positions in two-codon series) were analyzed independently for the two types of substitutions. The NSMC test was designed to account for differences in the nucleotide compositions and the frequencies of substitutions in synonymous and non-synonymous sites (non-synonymous sites were sampled to mimic the nucleotide composition in synonymous sites). With this normalization, the comparison of the number of hotspots, i.e., sites with at least two substitutions, between the samples of synonymous and non-synonymous (designated NSH and NNH, respectively) sites gives a measure of the skewness of the distribution of mutations (Fig. 1). Monte Carlo simulations, repeated 100,000 times, were used to assess the statistical significance of differences between the distributions of hotspots in the synonymous and non-synonymous sites. Two alternative statistical hypotheses were tested: *H0 *– mutational bias (no difference between the distributions of hotspots in the synonymous and non-synonymous sites) and *H1 *– selectional bias (the distributions of hotspots in the synonymous and non-synonymous sites are different). The fraction of simulated sets in which NNH > NSH is the probability P(*H1*) of the rejection of *H0*. Large values of P(*H1*) (≥ 0.95) indicate that the hypothesis *H0 *is rejected and there is a significant excess of hotspots in non-synonymous sites.

Using the NSMC test, we detected a statistically significant excess of hotspots in non-synonymous sites in 50% of the tumors for which extensive mutational data was available (Table 1). When the data from all tumor types were pooled, the excess of hotspots in non-synonymous sites was highly significant: the null hypothesis, i.e., that the distributions of mutations in the synonymous and non-synonymous sites were identical, was rejected with P < 0.001 (Table 2). Since we accounted for differences in nucleotide compositions, mutational biases are not expected to differ between synonymous and non-synonymous positions. Thus, the greater skew of the mutation distribution in non-synonymous positions should be viewed as evidence of, primarily, selectional origin of the hotspots.

**Table 2:** Combined NSMC and NSCS test results for the *TP53 *somatic mutations spectra

Class ID	Sites	Mutations	Hotspots	P(*H1*)/χ^2^
**Class I^a^**				
Synonymous	157	390	76	
Non-synonymous	527	5710	385	***0.990/33.4***
Nonsense	78	529	59	0.023
**Class II^b^**				
Synonymous	172	391	97	
Non-synonymous	495	6109	379	***0.991/25.4***
Nonsense	72	629	55	0.018
**Class III^c^**				
Synonymous	217	781	151	
Non-synonymous	617	11819	474	***0.994/4.47***
Nonsense	88	1158	76	0.011

^a ^**Class I**: combined mutational spectrum of the tumor types for which *H0 *is rejected in comparison of synonymous vs. non-synonymous substitutions:brain, liver, lung, colon, mouth, larynx, ovary and skin.

^b ^**Class II**: combined mutational spectrum of the tumor types for which the test did not find enough evidence, probably, because of the small sample size: bladder, breast, prostate, esophagus, colorectal, hematopoietic, stomach, and pancreas.

^c ^**Class III**: combined Class I and Class II spectra.

Other designations are as in Table 1.

This conclusion was further supported by analysis of mutation spectra after removal of CpG dinucleotides, the most prominent mutational hotspots in the human genome [24,25]. Under this test, many hotspots in CpG sites overlapping arginine, glycine and valine codons were removed but the selection hypothesis was nevertheless supported for several tissues and for the combined spectrum (Additional file 1). Furthermore, the results of the NSMC test performed before or after removal of the CpG sites did not depend on the threshold used for hotspot identification (Additional file 2).

We also applied the NSMC test to compare the distributions of hotspots in nonsense and synonymous sites. An excess of hotspots in nonsense sites would be indicative of positive selection for loss of p53 function. A significant excess of hotspots in nonsense sites was detected only in colorectal cancers as opposed to 8 of the 16 analyzed tumor types in which hotspots non-randomly associated with non-synonymous sites were identified (Table 1). The difference between the excess of hotspots in non-synonymous sites and the excess of hotspots in nonsense sites was statistically significant (P = 0.015 by the Fisher's exact test). This observation is compatible with the notion that non-synonymous hotspots in p53 evolve under positive selection for gain of function.

We further tested the hypothesis of independence between the mutation class (hotspot vs. non-hotspot) and site class (non-synonymous vs. synonymous). The data for all analyzed spectra were represented as 2 × 2 contingency tables which were analyzed using the χ^2 ^test (hereinafter NSCS test, after Non-Synonymous Chi-Square). Using the NSCS test, we observed a significant excess of hotspots in non-synonymous sites compared to the expectation under the independence hypothesis. Thus, two independent statistical tests show that, in the spectra of somatic mutations in the *TP53 *gene from most tumors, the hotspots are highly non-randomly associated with non-synonymous sites. In a direct analogy to the classical Ka/Ks signature of positive selection [13], this preferential occurrence of hotspots in non-synonymous positions indicates that the hotspots result, mostly, from positive selection for new functions of the p53 protein.

Both the NSMC and the NSCS tests produced opposite results when applied to the available mutational spectra of three other tumor suppressor genes, *BRCA1*, *BRCA2*, and *p16*^*INK4a *^(Table 3) [26]. The hypothesis that hotspots are randomly distributed among synonymous and non-synonymous sites could not be rejected for these genes. This observation suggests that p53 might be unique among tumor suppressors in that its somatic evolution in many tumors involves intense positive selection for gain of function. Alternatively, however, it cannot be ruled out that the available mutation data for the other tumor suppressors is insufficient to detect statistically significant association of hotspots with non-synonymous sites.

**Table 3:** The NSMC and NSCS test results for *BRCA1*, *BRCA2*, and *p16 *genes

Class ID	Sites	Mutations	Hotspots	P(*H1*)/χ^2^
***BRCA1***				
Synonymous	33	142	16	
Non-synonymous	356	1842	170	0.204/0.006
Nonsense	141	704	74	0.079
***BRCA2***				
Synonymous	55	103	20	
Non-synonymous	797	4871	352	0.071/1.2
Nonsense	179	844	79	0.018
***p16***				
Synonymous	40	63	13	
Non-synonymous	171	321	65	0.388/0.423
Nonsense	18	162	14	0.070

The data for combined spectra from all tumor types are shown. Other designations are as in Table 1.

We also developed a third statistical test (hereinafter NSB test, after Non-Synonymous Binomial) to identify non-synonymous substitution hotspots (analyzed, for this purpose, at the level of codons), i.e., those with a statistically significant excess of non-synonymous substitutions over the random expectation. The expected numbers of non-synonymous and synonymous substitutions were calculated using a Monte-Carlo simulation procedure, which was repeated 1,000 times for each codon. Each step involved random shuffling of transitions and transversions among the three positions of a codon. The statistical significance of the observed excess of the number of the detected non-synonymous substitution hotspots over the random expectation was assessed using the binomial test and the Bonferroni correction for multiple tests.

The NSB test revealed from 1 (*p16*) to 59 (*TP53*) hotspots non-randomly associated with non-synonymous sites in each of the tumor suppressors (Table 4). Thus, it appears that positive selection might affect not only somatic evolution of p53 but also that of other tumor suppressors albeit, seemingly, to a lesser extent. The failure of the NSMC and NSCS tests to detect the signature of positive selection in genes other than p53 could be due to the fact that these tests require a large number of synonymous substitutions which is currently available only for p53. Alternatively, however, it cannot be ruled out that synonymous substitutions are underrepresented in the databases for BRCA1, BRCA2, and p16. Such an artifact would affect the NSB test, potentially resulting in false-positives, but not the NSMC or the NSCS tests. Expanded compendia of somatic mutations for these genes and thorough database curation are critical for a reliable assessment of the contribution of positive selection to their evolution in tumors. Even the largest available database of somatic mutations, that for p53, is not large enough for some statistical experiments. For example, we were unable to apply our tests to G>T substitutions in lung tumors [18-20] because only a few unique synonymous G>T substitutions associated with lung tumors were found in the p53 database, whereas the non-synonymous G>T mutations are the most frequent type of substitutions in lung tumors [14,18-20]. Furthermore, more data on somatic mutations is required to explore the effect of nucleotide context other than that of CpG that was examined here.

**Table 4:** Hotspots non-randomly associated with non-synonymous sites in tumor suppressor genes according to the NSB test

Gene	Total number of codons	Number of non-synonymous hotspots
*TP53*	394	59
*BRCA1*	1864	12
*BRCA2*	3419	27
*p16*	157	1

The hotspots are listed in Additional file 3

It should be emphasized that, although we detected a highly statistically significant association of non-synonymous sites with hospots, in particular, for p53, the results of the present analysis do not allow us to assign any individual mutation to the gain-of-function or loss-of-function category. Nevertheless, these results can be used for devising experimental studies of gain-of-function by tumor suppressors mutated in specific sites (hotspots) and/or specific tumor types with particularly strong evidence of positive selection.

## Conclusion

The previous computational analysis of the tumor-specific mutation spectra of the *TP53 *gene has suggested that positive selection made a substantial contribution to the evolution of this gene during tumorigenesis [14,15]. Here, we show that positive selection, as opposed to mutational biases, is, largely, responsible for the formation of hotspots in the *TP53 *gene (of course, this does not rule out the existence of true mutational hotspots and their substantial role in tumorigenesis; we only show that such hotspots are in the minority). This finding is compatible with the previous observations that hotspots are located primarily in highly conserved, functionally important regions of p53 [14,17]. Together, the results of computational analyses of the mutational spectra strongly support the crucial role of gain-of-function in the tumorigenic evolution of p53, which agrees with the results of several experimental studies [4-10], in particular, the recent work on mouse models of the Li-Fraumeni syndrome [11,12]. It has been shown that hotspots are tumor-specific and, furthermore, include both residues that are directly involved in DNA-binding and residues that are important for maintaining the conformation of the p53 protein [14]. Thus, along with loss of function mutations, tumorigenesis might involve positive selection for a diverse set of novel activities of p53. The present analysis also yielded preliminary evidence of the role of positive selection in the evolution of BRCA1, BRCA2, and p16, suggesting that mutational gain-of-function in tumor suppressors might be a widespread and important aspect of tumorigenesis and blurring the boundary between tumor suppressors and oncogenes.

## Materials and methods

The *TP53 *mutation data were from the IARC database [27]. Sixteen tumor types were analyzed (the IARC "short topology" keyword = "BLADDER, BRAIN, BREAST, LIVER, LUNG, PANCREAS, OVARY, PROSTATE, COLON, COLORECTAL, ESOPHAGUS, HEMATOPOIETIC, LARYNX, MOUTH, SKIN, STOMACH"). The *p16*, *BRCA1 *and the *BRCA2 *mutation data were from the databases of mutation spectra in the *p16 *gene [28] and the *BRCA1 *and *BRCA2 *genes [29]. The statistical tests were implemented as *ad hoc *programs written in C++ or Perl.

## Reviewers' comments

### Reviewer's report 1

Sandor Pongor, International Centre for Genetic Engineering and Biotechnology, Padriciano 99, I-34012, Trieste, Italy

Detecting positive selection at the DNA sequence level is of substantial interest in view of the role it may play in pathogenic events. Glazko and coworkers show, using three statistical tests developed for the purpose, that the mutational hotspots of the TP53 gene evolve by positive selection. In view of the general interest of the topic I sought the advice of Dr. Lawrence Banks who is a biologist working on p53 mutations. We both felt that the calculations are thoroughly planned and the results support the main message of the paper.

The presentation of the paper could however be improved with special respect to the wide audience of Biology Direct. The most important criticism is that p53 mutations can lead both to inactivation and/or to GOF type changes, and these two groups may need to be analyzed separately. Currently, the reader may not see clearly if positive selection was found only in the case of GOF-type mutations or also in the case of inactivating mutations.

**Author response**: *We agree that it should be stated with full clarity that both gain-of-function and loss-of-function mutations in tumor suppressors, in particular, p53, are important. Therefore such changes have been made in several places in the manuscript; in particular, see the last paragraph in the Results and Discussion section*.

Minor points:

- In the Background section it might be useful to add 1) a brief description of mutation types found in p53 as well as their biological roles; and 2) a paragraph describing the mathematical approaches to detecting positive selection. These sections may help the reader in understanding what has been done and what is being accomplished in this work.

**Author response**: *We decided not to expand the Background section because both issues are already addressed there, even if briefly, and the reader interested in methods for detecting positive selection is referred to Ref*. [13].

- It may be useful to carry out the statistics on subgroups (GOF or inactivation)

**Author response**: *Some statistics on this point is available in Ref*. [16] (Table 1). *However, it has to be realized that, although we detect the statistical excess of non-synonymous over synonymous substitutions, the tests describe here, by themselves, do not allow us to assign an individual substitution to the gain-of-function or loss of function category. Again, an attempt to address this issue is given in Ref*. [16]*but the number of mutations for which the distinction could be made is quite small. We make comments to that effect in the revised discussion*.

- In order to show the strength of the statistical methods presented here it might be useful to consider tests similar to those described by Jianzhi Zhang (Mol. Biol. Evol. 21(7): 1332–1339, 2004).

**Author response**: *The statistical analysis presented here was done within a very different conceptual framework from that described by Zhang (maximum likelihood models). The present tests employed the multiple test (Bonferroni) correction and, accordingly, were highly conservative*.

### Reviewer's report 2

Christopher Lee, Department of Chemistry, University of California-Los Angeles, Los Angeles, CA, USA

This paper extends the authors' previous work indicating evidence of positive selection in p53 "hotspot" mutations, to show that non-synonymous mutations show a significantly greater tendency to cluster (in "hotspots") than do synonymous mutations, even when some mutational biases are taken into account. This work addresses an important biomedical question, and provides an advance, albeit incremental. I do have some questions which might benefit from further analysis by the authors:

1. Both in the abstract and introduction, the authors emphasize the importance of taking into consideration the effect of "nucleotide context" on mutational bias, as a motivation for this study. However, as I understand it, this study takes into consideration nucleotide composition (i.e. frequency of single nucleotides), not nucleotide context (e.g. frequency of nucleotide triplets, to consider the effect of one adjacent nucleotide on either side of the nucleotide under study). Since nucleotide context can have large effects on mutation rate (e.g. CpG effects), this is an important issue. For the very reasons that the authors articulated in their Introduction, many readers will expect direct tests of whether nucleotide context affects the authors' results.

The difficulty, of course, is that it is harder to match nucleotide context (e.g. triplet frequencies, 64 different numbers) than nucleotide composition (just 4 numbers). The NSMC procedure would probably not be able to construct samples with matching triplet frequencies, without some modifications. One possible solution would be to include ALL sites (including unmutated sites, instead of just sites where mutations were observed) in the analysis. First, generate a random sample of synonymous sites (a specific number of sites, with a specific triplet profile, and a specific number of observed mutations).

Now generate a random sample of non-synonymous sites of the same size, with the same triplet profile. Finally, generate equal-sized random samples of mutations from each set of sites, and analyze the number of "hotspots" as in the NSMC method. Including non-mutated sites in this sampling process should make it possible to match the triplet profiles between the syn vs. non-syn samples, and I don't see a reason why non-mutated sites should be excluded.

If such analysis is practical I think it could greatly strengthen the paper, by directly addressing the question of nucleotide context. At any rate, the existing analysis in the manuscript should be clearly described as testing "nucleotide composition" not "nucleotide context", and the difference between these should be emphasized. The authors should point out that even if composition is controlled for, nucleotide context could have large effects on mutation rate, so the current results should be interpreted with some caution.

**Author response**: *The CpG effects have been accounted for in the NSMC test; to emphasize this, we mention this control in the revised abstract. However, the currently available data on somatic mutations is insufficient to examine other, subtler effects of the nucleotide context. As for including non-mutated sites, we were concerned that this approach could lead to uncontrollable increase in the error rate due to the different and unknown intrinsic mutation rates of different sites*.

2. The NSMC analysis, while conceptually simple, needs to be described in more detail, in the Methods section. Currently, there is only an outline of NSMC, presented in the Results section, which leaves out many details (e.g. sampling with replacement or without replacement? I assume the latter), such that one could not replicate the calculation with any confidence that equivalent results would be obtained from the same input data.

**Author response**: *Indeed, sampling without replacement was employed, and this is mentioned in the revised legend to *Figure 1. *Otherwise, however, we felt that the description of the test was sufficient for reproduction*.

3. The manuscript frequently uses the term "positive selection", in a way that sometimes seems like a catch-all name for any significant divergence from the purely "mutational" process represented by synonymous sites. This may confuse readers who think of positive selection in terms of the very specific meaning Ka/Ks > 1, since that is not what this paper shows. Instead, the NNH>NSH "more hotspots" criterion gets at a somewhat different issue, namely the clustering of observed mutations at certain sites ("hotspots").

First, it should be noted that such clustering could be produced without Ka/Ks>1. For example, if most codons had Ka/Ks = 0.1, and a few sites had Ka/Ks = 1, this also could give rise to more "hotspots" compared with the synonymous sample (where no variability in selection occurs from site to site). Indeed, even if Ka/Ks = 1 everywhere, the fact that there are typically twice as many non-synonymous mutations than synonymous mutations at each codon could in principle give NNH>NSH. I think the authors should address this issue in the manuscript, either by providing control tests showing that their results cannot be explained by such models, and/or by mentioning such issues in the Discussion.

Second, the authors may want to replace a number of occurrences of the phrase "positive selection" with something more precise for their results, e.g. "selection for non-synonymous mutations at specific sites (hot spots), relative to their less frequent occurrence at other non-synonymous sites or at synonymous sites"; or just "evidence of selective pressure at hotspots". When the authors really want to use the phrase "positive selection", it would be useful to cite direct evidence that Ka/Ks > 1 for at least a subset of the sites.

**Author response**: *We already know that, at least in the case of somatic mutations of p53, Ka/Ks >> 1 *(Table 1*and Ref*. [14]) *which implies positive selection in the traditional sense. In this paper, we addressed a specific issue of origin of hotspots using different tests, within the "selection vs. mutation" framework. We believe that the NSB test adequately tests the hypothesis that "...Ka/Ks > 1 for at least a subset of the sites"*.

4. Since I'm not in the p53 field, it's unclear to me how cancer researchers can make use of the specific data presented in this paper. Perhaps the authors could add some further discussion of this to the paper.

**Author response**: *The last paragraph of the revised Results and Discussion section addresses this issue*.

### Reviewer's report 3

Mikhail Blagosklonny, Cancer Center, Ordway Research Institute, Albany, NY, USA

This study has demonstrated a selective advantage for hot spot p53 mutants compared with rare mutants. This has a biological meaning. p53 proteins form tetramers. Mutant p53 can either inactivate wt p53 or complement mutant p53, depending of particular mutation. Also, mutant p53 interacts with p63 and p73, thus modulating their functions. Similarly, the distinction between tumor suppressors and oncogenes might be blurred for p63 and p73, see: Mills AA. p63: oncogene or tumor suppressor? Curr Opin Genet Dev. 2005 Dec 13; in press.

**Author response**: *Unfortunately, large collections of mutations are unavailable for either p63 or p73*.

## Authors' contributions

IBR and EVK incepted the study and proposed the general principles of the tests for positive selection; GVG and IBR implemented the tests and performed the data analysis; VNB contributed to the statistical analysis of the results; EVK wrote the manuscript which was read, edited, and approved by all authors.

## Supplementary Material

Additional File 1 NSMC test results for the p53 spectra with CpG sites removed (*H0*: mutational bias; *H1*: selectional bias). Click here for file

Additional File 2 NSMC test results for combined spectra for the 4 analyzed tumor suppressors^a^. Click here for file

Additional File 3 Hotspots non-randomly associate with non-synonymous sites according to the NSB test. Click here for file

## References

[B1] Hansen R, Oren M (1997). p53; from inductive signal to cellular effect. Curr Opin Genet Dev.

[B2] Levine AJ (1997). p53, the cellular gatekeeper for growth and division. Cell.

[B3] Agarwal ML, Taylor WR, Chernov MV, Chernova OB, Stark GR (1998). The p53 network. J Biol Chem.

[B4] Guimaraes DP, Hainaut P (2002). TP53: a key gene in human cancer. Biochimie.

[B5] Lane DP, Benchimol S (1990). p53: oncogene or anti-oncogene?. Genes Dev.

[B6] Dittmer D, Pati S, Zambetti G, Chu S, Teresky AK, Moore M, Finlay C, Levine AJ (1993). Gain of function mutations in p53. Nat Genet.

[B7] Hsiao M, Low J, Dorn E, Ku D, Pattengale P, Yeargin J, Haas M (1994). Gain-of-function mutations of the p53 gene induce lymphohematopoietic metastatic potential and tissue invasiveness. Am J Pathol.

[B8] Blagosklonny MV (2000). p53 from complexity to simplicity: mutant p53 stabilization, gain-of-function, and dominant-negative effect. Faseb J.

[B9] Pugacheva EN, Ivanov AV, Kravchenko JE, Kopnin BP, Levine AJ, Chumakov PM (2002). Novel gain of function activity of p53 mutants: activation of the dUTPase gene expression leading to resistance to 5-fluorouracil. Oncogene.

[B10] Resnick MA, Inga A (2003). Functional mutants of the sequence-specific transcription factor p53 and implications for master genes of diversity. Proc Natl Acad Sci U S A.

[B11] Olive KP, Tuveson DA, Ruhe ZC, Yin B, Willis NA, Bronson RT, Crowley D, Jacks T (2004). Mutant p53 gain of function in two mouse models of Li-Fraumeni syndrome. Cell.

[B12] Lang GA, Iwakuma T, Suh YA, Liu G, Rao VA, Parant JM, Valentin-Vega YA, Terzian T, Caldwell LC, Strong LC, El-Naggar AK, Lozano G (2004). Gain of function of a p53 hot spot mutation in a mouse model of Li-Fraumeni syndrome. Cell.

[B13] Hurst LD (2002). The Ka/Ks ratio: diagnosing the form of sequence evolution. Trends Genet.

[B14] Glazko GV, Koonin EV, Rogozin IB (2004). Mutation hotspots in the p53 gene in tumors of different origin: correlation with evolutionary conservation and signs of positive selection. Biochim Biophys Acta.

[B15] Koonin EV, Rogozin IB, Glazko GV (2005). p53 gain-of-function: tumor biology and bioinformatics come together. Cell Cycle.

[B16] Gorlov IP, Gorlova OY, Amos CI (2005). Predicting the oncogenicity of missense mutations reported in the International Agency for Cancer Research (IARC) mutation database on p53. Hum Mutat.

[B17] Walker DR, Bond JP, Tarone RE, Harris CC, Makalowski W, Boguski MS, Greenblatt MS (1999). Evolutionary conservation and somatic mutation hotspot maps of p53: correlation with p53 protein structural and functional features. Oncogene.

[B18] Denissenko MF, Pao A, Tang M, Pfeifer GP (1996). Preferential formation of benzo[a]pyrene adducts at lung cancer mutational hotspots in P53. Science.

[B19] Hainaut P, Pfeifer GP (2001). Patterns of p53 G-->T transversions in lung cancers reflect the primary mutagenic signature of DNA-damage by tobacco smoke. Carcinogenesis.

[B20] Rodin SN, Rodin AS (2002). On the origin of p53 G:C --> T:A transversions in lung cancers. Mutat Res.

[B21] Rogozin IB, Pavlov YI, Bebenek K, Matsuda T, Kunkel TA (2001). Somatic mutation hotspots correlate with DNA polymerase eta error spectrum. Nat Immunol.

[B22] Pham P, Bransteitter R, Petruska J, Goodman MF (2003). Processive AID-catalysed cytosine deamination on single-stranded DNA simulates somatic hypermutation. Nature.

[B23] Kotani A, Okazaki IM, Muramatsu M, Kinoshita K, Begum NA, Nakajima T, Saito H, Honjo T (2005). A target selection of somatic hypermutations is regulated similarly between T and B cells upon activation-induced cytidine deaminase expression. Proc Natl Acad Sci U S A.

[B24] Cooper DN, Youssoufian H (1988). The CpG dinucleotide and human genetic disease. Hum Genet.

[B25] Rogozin IB, Pavlov YI (2003). Theoretical analysis of mutation hotspots and their DNA sequence context specificity. Mutat Res.

[B26] Vogelstein B, Kinzler KW (2004). Cancer genes and the pathways they control. Nat Med.

[B27] Olivier M, Eeles R, Hollstein M, Khan MA, Harris CC, Hainaut P (2002). The IARC TP53 database: new online mutation analysis and recommendations to users. Hum Mutat.

[B28] p16 mutation database [http://srs6.ebi.ac.uk/srs6bin/cgi-bin/wgetz?-newId+-page+LibInfo+-lib+P16].

[B29] An Open Access On-Line Breast Cancer Mutation Data Base [(http://research.nhgri.nih.gov/bic/].

