# Supplementary material for: Mutational hotspots in the TP53 gene and, possibly, other tumor suppressors evolve by positive selection
Source: Biol Direct. 2006 Jan 31;1:4. doi: 10.1186/1745-6150-1-4 (PMC1403748; doi:10.1186/1745-6150-1-4)
Supplement: Additional File 1 — NSMC test results for the p53 spectra with CpG sites removed (H0: mutational bias; H1: selectional bias). [file 1745-6150-1-4-S1.doc]

Supplementary Table 1. NSMC test results for the p53 spectra with CpG sites removed (*H0*: mutational bias; *H1*: selectional bias).

|  | Sites | | Mutations | Hotspots | | P (*H1*) | |
| --- | --- | --- | --- | --- | --- | --- | --- |
| ***Bladder*** | | | | | | | |
| Synonymous | 60 | | 79 | 12 | |  | |
| Non-synonymous | 229 | | 544 | 85 | | 0.086 | |
| ***Brain*** | | | | | | | |
| Synonymous | 18 | | 21 | 2 | |  | |
| Non-synonymous | 184 | | 609 | 87 | | 0.024 | |
| ***Breast*** | | | | | | | |
| Synonymous | 73 | | 103 | 18 | |  | |
| Non-synonymous | 279 | | 1277 | 161 | | ***0.989*** | |
| ***Liver*** | | | | | | | |
| Synonymous | 33 | | 40 | 4 | |  | |
| Non-synonymous | 188 | | 680 | 93 | | ***0.987*** | |
| ***Lung*** | | | | | | | |
| Synonymous | 54 | | 83 | 16 | |  | |
| Non-synonymous | 299 | | 1465 | 181 | | ***0.994*** | |
| ***Pancreas*** | | | | | | | |
| Synonymous | 10 | | 12 | 1 | |  | |
| Non-synonymous | 101 | | 183 | 27 | | 0.062 | |
| ***Ovary*** | | | | | | | |
| Synonymous | 18 | | 19 | 1 | |  | |
| Non-synonymous | 183 | | 678 | 95 | | 0.026 | |
| ***Prostate*** | | | | | | | |
| Synonymous | 26 | | 36 | 7 | |  | |
| Non-synonymous | 107 | | 169 | 35 | | 0.030 | |
| ***Colon*** | | | | | | | |
| Synonymous | 20 | | 25 | 4 | |  | |
| Non-synonymous | 160 | | 467 | 65 | | 0.006 | |
| ***Colorectal*** | | | | | | | |
| Synonymous | 23 | | 26 | 3 | |  | |
| Non-synonymous | 193 | | 901 | 116 | | 0.000 | |
| ***Esophagus*** | | | | | | | |
| Synonymous | 24 | | 31 | 7 | |  | |
| Non-synonymous | 204 | | 927 | 112 | | 0.032 | |
| ***Hematopoietic*** | | | | | | | |
| Synonymous | 24 | | 26 | 2 | |  | |
| Non-synonymous | 189 | | 648 | 94 | | 0.045 | |
| ***Larynx*** | | | | | | | |
| Synonymous | 6 | | 7 | 1 | |  | |
| Non-synonymous | 104 | | 219 | 39 | | 0.000 | |
| ***Mouth*** | | | | | | | |
| Synonymous | 27 | | 38 | 8 | |  | |
| Non-synonymous | 164 | | 370 | 74 | | ***0.975*** | |
| ***Skin*** | | | | | | | |
| Synonymous | 62 | | 134 | 12 | |  | |
| Non-synonymous | 233 | | 511 | 91 | | ***0.925*** | |
| ***Stomach*** | | | | | | | |
| Synonymous | 42 | | 61 | 14 | |  | |
| Non-synonymous | 184 | | 519 | 81 | | 0.136 | |
| ***All spectra combined*** | | | | | | | |
| Synonymous | 205 | 741 | | | 145 | |  |
| Non-synonymous | 591 | 10326 | | | 451 | | ***0.993*** |
