# Supplementary material for: Mutational hotspots in the TP53 gene and, possibly, other tumor suppressors evolve by positive selection
Source: Biol Direct. 2006 Jan 31;1:4. doi: 10.1186/1745-6150-1-4 (PMC1403748; doi:10.1186/1745-6150-1-4)
Supplement: Additional File 2 — NSMC test results for combined spectra for the 4 analyzed tumor suppressorsa. [file 1745-6150-1-4-S2.doc]

Supplementary Table 2. NSMC test results for combined spectra for the 4 analyzed tumor suppressors^a^.

| Class ID | Sites | Mutations | hs:μ≥3 | hs:μ≥4 | P(*H1*|μ≥3)/ P(*H1*|μ≥4) |
| --- | --- | --- | --- | --- | --- |
| ***TP53*** | | | | | |
| Synonymous | 217 | 781 | 113 | 72 |  |
| Non-synonymous | 617 | 11819 | 411 | 359 | ***0.989/0.984*** |
| Nonsense | 88 | 1158 | 65 | 52 | 0.009/0.007 |
| ***BRCA1*** | | | | | |
| Synonymous | 33 | 142 | 9 | 5 |  |
| Non-synonymous | 356 | 1842 | 120 | 83 | 0.195/0.146 |
| Nonsense | 141 | 704 | 52 | 38 | 0.065/0.004 |
| ***BRCA2*** | | | | | |
| Synonymous | 55 | 103 | 8 | 7 |  |
| Non-synonymous | 797 | 4871 | 215 | 169 | 0.069/0.069 |
| Nonsense | 179 | 844 | 51 | 31 | 0.005/0.003 |
| ***p16*** | | | | | |
| Synonymous | 40 | 63 | 5 | 4 |  |
| Non-synonymous | 171 | 321 | 31 | 14 | 0.197/0.098 |
| Nonsense | 18 | 162 | 13 | 10 | 0.065/0.047 |

^a^ A hotspot (hs) was defined as a site with the number of substitutions equal to or greater than μ = 3 or μ = 4 (*H0*: mutational bias; *H1*: selectional bias).
