# Supplementary material for: Mutational hotspots in the TP53 gene and, possibly, other tumor suppressors evolve by positive selection
Source: Biol Direct. 2006 Jan 31;1:4. doi: 10.1186/1745-6150-1-4 (PMC1403748; doi:10.1186/1745-6150-1-4)
Supplement: Additional File 3 — Hotspots non-randomly associate with non-synonymous sites according to the NSB test. [file 1745-6150-1-4-S3.doc]

Supplementary Table 3. Hotspots non-randomly associate with non-synonymous sites according to the NSB test.

| Codon number | # syn. observed | | #non-syn. observed | | #  nonsense observed | | # syn. expected | | #non-syn. expected | | | P_value |  |
| --- | --- | --- | --- | --- | --- | --- | --- | --- | --- | --- | --- | --- | --- |
| TP53 |  | |  | |  | |  | |  | | |  |  |
| 273 | 9 | | 1237 | | 0 | | 439.6 | | 806.4 | | | 2.94E-216 |  |
| 248 | 32 | | 1267 | | 0 | | 462.8 | | 836.2 | | | 2.37E-193 |  |
| 175 | 6 | | 943 | | 0 | | 308.2 | | 640.8 | | | 1.87E-149 |  |
| 282 | 16 | | 513 | | 0 | | 209.3 | | 319.7 | | | 3.58E-89 |  |
| 245 | 16 | | 595 | | 0 | | 174.4 | | 436.6 | | | 4.29E-65 |  |
| 278 | 2 | | 213 | | 0 | | 75.8 | | 139.2 | | | 1.67E-37 |  |
| 179 | 5 | | 292 | | 0 | | 80.4 | | 216.6 | | | 2.70E-33 |  |
| 249 | 8 | | 501 | | 0 | | 89 | | 420 | | | 1.45E-31 |  |
| 220 | 0 | | 276 | | 3 | | 62.6 | | 216.4 | | | 3.46E-31 |  |
| 176 | 1 | | 271 | | 4 | | 60.3 | | 215.7 | | | 6.14E-28 |  |
| 237 | 0 | | 169 | | 0 | | 45.2 | | 123.8 | | | 1.52E-23 |  |
| 241 | 5 | | 145 | | 0 | | 52.2 | | 97.8 | | | 3.75E-21 |  |
| 285 | 6 | | 153 | | 15 | | 51.3 | | 122.7 | | | 9.08E-17 |  |
| 238 | 4 | | 147 | | 5 | | 44 | | 112 | | | 1.04E-16 |  |
| 158 | 9 | | 201 | | 0 | | 52.8 | | 157.2 | | | 4.52E-16 |  |
| 193 | 2 | | 155 | | 0 | | 36.3 | | 120.7 | | | 1.40E-15 |  |
| 246 | 0 | | 115 | | 0 | | 29.4 | | 85.6 | | | 1.72E-15 |  |
| 280 | 4 | | 191 | | 7 | | 42.2 | | 159.8 | | | 4.44E-15 |  |
| 266 | 4 | | 140 | | 18 | | 42.5 | | 119.5 | | | 2.94E-14 |  |
| 234 | 2 | | 128 | | 13 | | 35 | | 108 | | | 1.32E-13 |  |
| 163 | 4 | | 148 | | 15 | | 40.2 | | 126.8 | | | 1.51E-13 |  |
| 275 | 3 | | 120 | | 2 | | 34.2 | | 90.8 | | | 1.53E-13 |  |
| 205 | 1 | | 127 | | 7 | | 30.4 | | 104.5 | | | 2.36E-13 |  |
| 141 | 6 | | 109 | | 9 | | 39.2 | | 84.8 | | | 3.38E-12 |  |
| 242 | 6 | | 157 | | 4 | | 38.8 | | 128.2 | | | 3.82E-12 |  |
| 286 | 2 | | 106 | | 18 | | 32.1 | | 93.9 | | | 1.14E-11 |  |
| 151 | 19 | | 149 | | 0 | | 56.5 | | 111.5 | | | 2.19E-11 |  |
| 258 | 1 | | 96 | | 16 | | 28.3 | | 84.7 | | | 2.32E-11 |  |
| 272 | 7 | | 126 | | 0 | | 37.1 | | 95.9 | | | 2.41E-11 |  |
| 213 | 3 | | 64 | | 205 | | 104.4 | | 167.6 | | | 1.02E-10 |  |
| 135 | 11 | | 147 | | 6 | | 44 | | 120 | | | 2.08E-10 |  |
| 152 | 14 | | 103 | | 0 | | 44.8 | | 72.2 | | | 2.14E-10 |  |
| 244 | 14 | | 165 | | 0 | | 47.2 | | 131.8 | | | 2.69E-10 |  |
| 132 | 2 | | 128 | | 2 | | 25.6 | | 106.4 | | | 3.44E-10 |  |
| 270 | 2 | | 84 | | 0 | | 24 | | 62 | | | 3.50E-10 |  |
| 181 | 3 | | 75 | | 0 | | 24 | | 54 | | | 2.55E-09 |  |
| 194 | 5 | | 87 | | 0 | | 27.5 | | 64.5 | | | 5.15E-09 |  |
| 173 | 14 | | 157 | | 0 | | 43.6 | | 127.4 | | | 6.61E-09 |  |
| 216 | 5 | | 75 | | 0 | | 24.5 | | 55.5 | | | 9.75E-08 |  |
| 161 | 10 | | 87 | | 0 | | 32.2 | | 64.8 | | | 1.47E-07 |  |
| 239 | 3 | | 89 | | 0 | | 20.1 | | 71.9 | | | 4.19E-07 |  |
| 195 | 12 | | 95 | | 0 | | 34.2 | | 72.8 | | | 5.05E-07 |  |
| 157 | 15 | | 179 | | 0 | | 39.3 | | 154.7 | | | 1.35E-06 |  |
| 214 | 3 | | 68 | | 0 | | 17.9 | | 53.1 | | | 2.63E-06 |  |
| 159 | 11 | | 87 | | 0 | | 30.6 | | 67.4 | | | 3.05E-06 |  |
| 134 | 0 | | 33 | | 0 | | 10.2 | | 22.8 | | | 5.10E-06 |  |
| 236 | 5 | | 81 | | 14 | | 23.9 | | 76.1 | | | 8.08E-06 |  |
| 276 | 3 | | 65 | | 0 | | 16.7 | | 51.3 | | | 9.91E-06 |  |
| 190 | 5 | | 43 | | 0 | | 18.6 | | 29.4 | | | 1.30E-05 |  |
| 138 | 10 | | 71 | | 0 | | 26.8 | | 54.2 | | | 1.69E-05 |  |
| 243 | 0 | | 33 | | 0 | | 8.8 | | 24.2 | | | 3.79E-05 |  |
| 218 | 1 | | 39 | | 0 | | 11.1 | | 28.9 | | | 3.88E-05 |  |
| 215 | 2 | | 58 | | 0 | | 13.4 | | 46.6 | | | 4.09E-05 |  |
| 274 | 3 | | 62 | | 0 | | 15.1 | | 49.9 | | | 5.05E-05 |  |
| 110 | 0 | | 34 | | 0 | | 8.5 | | 25.5 | | | 5.80E-05 |  |
| 279 | 4 | | 49 | | 0 | | 16.1 | | 36.9 | | | 5.82E-05 |  |
| 169 | 0 | | 28 | | 0 | | 7.9 | | 20.1 | | | 8.93E-05 |  |
| 277 | 6 | | 72 | | 6 | | 20.8 | | 63.2 | | | 1.03E-04 |  |
| 251 | 3 | | 60 | | 0 | | 14.3 | | 48.7 | | | 1.11E-04 |  |
| p16 |  | |  | |  | |  | |  | | |  |  |
| 114 | 0 | | 19 | | 0 | | 8.8 | | 9.7 | | | 4.78E-06 |  |
| BRCA1 |  | |  | |  | |  | |  | | |  |  |
| 1347 | | 0 | | 142 | | 0 | | 38.5 | | 103.5 | 3.18E-20 | | |
| 1628 | | 0 | | 44 | | 0 | | 21.8 | | 22.2 | 7.82E-14 | | |
| 841 | | 0 | | 65 | | 0 | | 21.8 | | 37 | 8.33E-14 | | |
| 61 | | 0 | | 118 | | 0 | | 24.4 | | 89.5 | 4.49E-13 | | |
| 496 | | 0 | | 60 | | 0 | | 17 | | 40.3 | 6.42E-10 | | |
| 1008 | | 0 | | 68 | | 0 | | 17.2 | | 49.8 | 1.70E-09 | | |
| 772 | | 0 | | 27 | | 0 | | 13.9 | | 13.1 | 3.59E-09 | | |
| 356 | | 0 | | 52 | | 1 | | 15.4 | | 37.5 | 1.67E-08 | | |
| 1637 | | 0 | | 37 | | 0 | | 12.7 | | 20.8 | 2.20E-08 | | |
| 486 | | 0 | | 18 | | 0 | | 8.9 | | 9.1 | 4.39E-06 | | |
| 1652 | | 0 | | 37 | | 0 | | 10.2 | | 26.2 | 5.14E-06 | | |
| 871 | | 0 | | 23 | | 0 | | 7.7 | | 13.2 | 2.42E-05 | | |
| BRCA2 | |  | |  | |  | |  | |  |  | | |
| 2490 | | 0 | | 237 | | 0 | | 119.2 | | 117.8 | 1.23E-72 | | |
| 3414 | | 0 | | 156 | | 0 | | 48.4 | | 107.6 | 7.04E-26 | | |
| 505 | | 0 | | 84 | | 0 | | 41.5 | | 42.5 | 1.48E-25 | | |
| 42 | | 0 | | 140 | | 0 | | 43.3 | | 96.7 | 3.36E-23 | | |
| 3412 | | 0 | | 109 | | 0 | | 33.7 | | 75.3 | 3.14E-18 | | |
| 384 | | 0 | | 98 | | 0 | | 27.2 | | 62 | 3.19E-16 | | |
| 2034 | | 0 | | 95 | | 0 | | 26.4 | | 60.2 | 1.01E-15 | | |
| 2108 | | 0 | | 108 | | 0 | | 25.2 | | 79.3 | 1.11E-13 | | |
| 2440 | | 0 | | 79 | | 0 | | 24.6 | | 54.4 | 1.52E-13 | | |
| 2856 | | 0 | | 131 | | 0 | | 21.7 | | 109.3 | 5.16E-11 | | |
| 2116 | | 0 | | 65 | | 0 | | 19.8 | | 45.2 | 5.24E-11 | | |
| 598 | | 0 | | 62 | | 0 | | 19.4 | | 42.6 | 8.14E-11 | | |
| 2728 | | 0 | | 87 | | 0 | | 19.3 | | 65.6 | 1.77E-10 | | |
| 2285 | | 0 | | 59 | | 0 | | 18.5 | | 40.5 | 2.39E-10 | | |
| 2944 | | 0 | | 115 | | 0 | | 16.8 | | 86.6 | 1.44E-09 | | |
| 935 | | 0 | | 82 | | 0 | | 17.4 | | 63.2 | 2.07E-09 | | |
| 929 | | 0 | | 29 | | 0 | | 14.4 | | 14.6 | 2.23E-09 | | |
| 2515 | | 0 | | 53 | | 0 | | 14.3 | | 34.1 | 8.47E-09 | | |
| 3244 | | 0 | | 73 | | 0 | | 15.8 | | 55.5 | 1.12E-08 | | |
| 784 | | 0 | | 47 | | 0 | | 14.2 | | 32.8 | 4.29E-08 | | |
| 2466 | | 0 | | 48 | | 0 | | 12.9 | | 30.7 | 4.82E-08 | | |
| 1902 | | 0 | | 60 | | 0 | | 13.5 | | 45.2 | 1.49E-07 | | |
| 1420 | | 0 | | 190 | | 0 | | 11.5 | | 143 | 4.34E-07 | | |
| 1529 | | 0 | | 53 | | 0 | | 11.8 | | 40.1 | 1.18E-06 | | |
| 2835 | | 0 | | 18 | | 1 | | 9.6 | | 9.3 | 2.94E-06 | | |
| 655 | | 0 | | 107 | | 0 | | 10.9 | | 88.3 | 4.05E-06 | | |
| 3013 | | 0 | | 33 | | 0 | | 9.3 | | 20.9 | 5.14E-06 | | |

The threshold value for each spectrum was defined using the Bonferroni correction for multiple tests.
